# Comparison of outcomes between emergent-start and planned-start peritoneal dialysis in incident ESRD patients: a prospective observational study

**DOI:** 10.1186/s12882-017-0764-6

**Published:** 2017-12-11

**Authors:** Wen-Yi Li, Yi-Cheng Wang, Shang-Jyh Hwang, Shih-Hua Lin, Kwan-Dun Wu, Yung-Ming Chen

**Affiliations:** 1 Renal Division, Department of Internal Medicine, National Taiwan University Hospital, Yunlin Branch, Yunlin, Taiwan; 2 Renal Division, Department of Internal Medicine, National Taiwan University Hospital, College of Medicine, National Taiwan University, No. 7 Chung-Shan South Road, Taipei, 100 Taiwan; 3 Renal Division, Department of Internal Medicine, National Taiwan University Hospital, Taipei, Taiwan; 4 Division of Nephrology, Department of Internal Medicine, Kaohsiung Medical University Hospital, Faculty of Renal Care, College of Medicine, Kaohsiung Medical University, Kaohsiung, Taiwan; 5 Division of Geriatrics and Gerontology, Institute of Population Health Sciences, National Health Research Institutes, Miaoli, Taiwan; 6 Division of Nephrology, Department of Internal Medicine, Tri-Service General Hospital, National Defense Medical Center, Taipei, Taiwan

**Keywords:** Peritoneal dialysis, Emergent-start, Mortality

## Abstract

**Background:**

The clinical consequences of starting chronic peritoneal dialysis (PD) after emergent dialysis via a temporary hemodialysis (HD) catheter has rarely been evaluated within a full spectrum of treated end-stage renal disease (ESRD). We investigated the longer-term outcomes of patients undergoing emergent-start PD in comparison with that of other practices of PD or HD in a prospective cohort of new-onset ESRD.

**Methods:**

This was a 2-year prospective observational study. We enrolled 507 incident ESRD patients, among them 111 chose PD (43 planned-start, 68 emergent-start) and 396 chose HD (116 planned-start, 280 emergent-start) as the long-term dialysis modality. The logistic regression model was used to identify variables associated with emergent-start dialysis. The Kaplan–Meier survival analysis was used to determine patient survival and technique failure. The propensity score-adjusted Cox regression model was used to identify factors associated with patient outcomes.

**Results:**

During the 2-year follow-up, we observed 5 (4.5%) deaths, 15 (13.5%) death-censored technique failures (transfer to HD) and 3 (2.7%) renal transplantations occurring in the PD population. Lack of predialysis education, lower predialysis estimated glomerular filtration rate and serum albumin were predictors of being assigned to emergent dialysis initiation. The emergent starters of PD displayed similar risks of patient survival, technique failure and overall hospitalization, compared with the planned-start counterparts. By contrast, the concurrent planned-start and emergent-start HD patients with an arteriovenous fistula or graft were protected from early overall death and access infection-related mortality, compared with the emergent HD starters using a central venous catheter.

**Conclusions:**

In late-referred chronic kidney disease patients who have initiated emergent dialysis via a temporary HD catheter, post-initiation PD can be a safe and effective long-term treatment option. Nevertheless, due to the potential complications and cost concerns, such practice of PD initiation would better be replaced with a planned-start mode by employing more effective predialysis therapeutic education and timely catheter placement.

## Background

Peritoneal dialysis (PD) is a well-established modality of long-term dialysis treatment for newly discovered patients with end-stage renal disease (ESRD) [1, 2]. Most clinical practice guidelines recommend to place a PD catheter at least 2 weeks prior to the anticipated need of long-term PD treatment, i.e., planned-start PD [3]. However, patients may be forced to start PD treatment urgently due to intolerable uremic symptoms, even though the catheter has just been placed for less than 2 weeks [4]. Such practice of PD initiation, i.e., urgent-start PD, has recently gained resurgent momentum as an option of dialysis treatment especially for the late-referred CKD patients [5–7]. Indeed, there is accumulating evidence indicates that urgent-starters of PD exhibit a similar short-term patient survival and technique failure as the traditionally planned counterpart [8, 9].

Despite wide-spread promotion of planned-start dialysis therapy for patients with advanced chronic kidney disease (CKD), there remain patients who are referred ultra-late to the nephrologists, [4, 10] and acute dialysis has to be initiated via a temporary hemodialysis (HD) catheter due to life-threatening uremic emergency [11, 12]. After stabilization of vital conditions, most such patients tend to choose HD out of convenience as the long-term dialysis modality, [10, 13] although some may select post-initiation PD, i.e., emergent-start PD, as the treatment option. Compared to the above-mentioned urgent-start PD which is considered a safe and effective option of dialysis initiation, data are still limited regarding the impact of emergent-start PD in new-onset patients with ESRD [13]. To shed light on this issue, we undertook this observational study to make clear the longer-term outcomes of patients who underwent emergent-start PD in comparison with that of other practices of PD or HD in a prospective cohort of incident ESRD patients over a period of 2 years.

## Methods

### Study population

This observational study recruited incident ESRD patients who underwent first dialysis treatment via a permanent peritoneal or vascular access, either preformed without or created after emergent dialysis via a temporary HD catheter, between January 2009 and July 2011 from 3 academic medical centers. The study protocol was approved by the Research Ethics Committee of National Taiwan University Hospital (No. 200810042R), and the Institutional Review Boards of Tri-Service General Hospital (No. 070–05-114) and Kaohsiung Medical University Chung-Ho Memorial Hospital (No. 980077). Written informed consent was obtained from all participants before the start of the study.

### Group definition

The PD participants were divided into 2 groups according to the timing of PD catheter placement in relation to dialysis initiation: (a) the planned-start group received preemptive placement of a PD catheter and started first dialysis >2 weeks after the surgery; (b) the emergent-start group underwent post-initiation placement of a PD catheter after acute dialysis for uremic emergency via a temporary HD catheter.

Concurrent HD participants in the same cohort were included as the comparison group. The definition of grouping had been described previously [14]. Briefly, the planned starters commenced first HD via a preformed arteriovenous fistula (AVF, >4 weeks after the surgery) or arteriovenous graft (AVG, >2 weeks after the surgery) (planned-start HD-AVF/AVG); the emergent starters commenced maintenance HD via a post-initiation AVF or AVG (emergent-start HD-AVF/AVG), or a tunneled cuffed central venous catheter (CVC) (emergent-start HD-CVC) after acute dialysis for uremic emergency via a temporary HD catheter.

### Dialysis treatment

Decisions to initiate first dialysis in the planned starters or acute dialysis in the emergent starters were made by treating nephrologists based on regulations mandated by the National Health Insurance Administration of Taiwan as follows: (1) absolute indications, creatinine clearance (CCr) <5 ml/min or serum creatinine >10 mg/dl (884 μmol/l); (2) relative indications, CCr <15 ml/min or serum creatinine >6 mg/dl (530 μmol/l), plus the presence of fluid overload or other uremic emergency [15].

Patients with a PD catheter received either continuous ambulatory or automated PD, with a target weekly Kt/V of at least 1.7 using glucose-based PD solutions, and icodextrin-containing or amino acid-based PD solutions when appropriate [16]. In the meantime, patients with a permanent vascular access (AVF, AVG) or a tunneled cuffed CVC underwent thrice-weekly standard bicarbonate HD, with a target Kt/V of at least 1.2 using single-used dialyzers with high-efficient or high-flux membranes [17].

### Outcome measures

Baseline demographics, primary etiologies or comorbidities, participation in the predialysis education program, and biochemical data were documented at time of enrollment at each clinical site. Glomerular filtration rate (GFR) was estimated at the time of first dialysis (planned starters) or acute dialysis (emergent starters) by using the CKD Epidemiology Collaboration equation [18]. The primary endpoints were all-cause and cause-specific mortality. The secondary endpoints were technique failure (PD population) and overall hospitalization. All participants were followed over 2 years until death, kidney transplantation or the end of 2013.

### Statistical analysis

Statistical analyses were performed with IBM SPSS Statistics for Windows, version 22.0.0 (IBM Corp., Armonk, NY, USA) and Stata/SE for Windows, version 14 (StataCorp, LP, College Station, TX, USA). The distributional properties of data were expressed as mean ± standard deviation for continuous variables with a normal distribution or median (interquartile range) for those with a skewed distribution. For numerical data, Wilcoxon signed rank test was used for comparisons within groups, and Kruskal-Wallis test was used for comparisons between groups; for categorical variables with percentage (%), chi-squared or Fisher’s exact test was used. Multivariate logistic regression analysis was performed to determine the predictors for being assigned to emergent-start dialysis. The propensity score for each patient based on the probability of emergent dialysis initiation was then included in the analysis of Cox proportional hazards model. Unadjusted survival analyses of time to outcome measures were conducted using the Kaplan-Meier method and the log-rank test. The observed time started from the date of first dialysis treatment via a preformed AVF/AVG or PD catheter (planned starters), or a post-initiation PD catheter, AVF/AVG or tunneled cuffed CVC (emergent starters). For the analysis of overall survival in PD (or HD) patients, the event was death, and kidney transplantation or transfer to HD (or PD) was the censored observation. For the analysis of technique survival in PD patients, transfer to HD was the event, and censored observations included death and kidney transplantation. Multivariate time-dependent Cox proportional hazards models were constructed to determine independent variables associated with all-cause and access infection-related mortality. Dialysis modality or access type was treated as a time-dependent variable in the Cox regression model using the so-called counting process approach. It was defined by the time from baseline when a switch of the dialysis modality or access type occurred. The non-time-dependent variables considered in the regression analysis were demographics, primary etiologies or comorbidities, participation in the predialysis education program, and biochemical data. Validity of the Cox proportional hazard assumption in final models was checked by the log-minus-log graphical method. We also constructed competing risk regression models [19] for access infection-related mortality and overall hospitalization that treated death from other cause, and death and renal transplantation, respectively, as the competing events for estimating the risk. The results of Cox regression analysis were shown as hazards ratio (HR) or sub-hazards ratio (sub-HR) followed by 95% confidence intervals (CI). Significant differences were defined as *p* value less than 0.05.

## Results

### Baseline patient characteristics

**Fig. 1:**
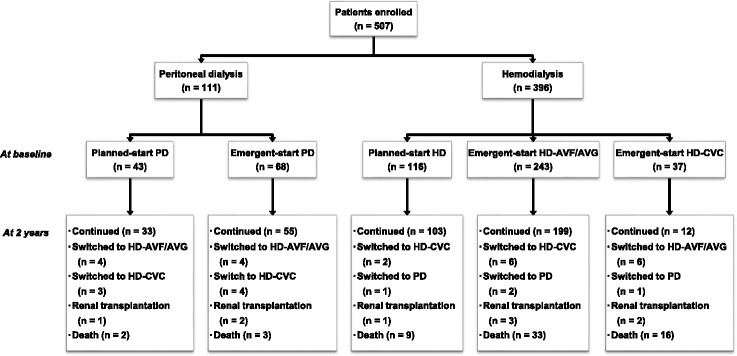
Flow diagram of all participants and their outcomes in the first two years after initiation of long-term dialysis therapy. HD, hemodialysis; PD, peritoneal dialysis; AVF, arteriovenous fistula; AVG, arteriovenous graft; CVC, central venous catheter

A total of 507 patients (mean age 61.8 years, male 56.8%) were enrolled in this study (Fig. 1). The baseline characteristics of these participants as categorized by dialysis modality (PD vs. HD) and initiation pattern (planned vs. emergent) are presented in Table 1. Among them, 111 (21.9%) were under PD and 396 (78.1%) were under HD. The most common primary cause for ESRD differed between PD (41.4% chronic glomerulonephritis) and HD (48.0% diabetic nephropathy) populations. There were no differences in comorbidities between emergent-start PD patients and their planned-start counterparts. In contrast, a history of congestive heart failure was higher in the emergent-start HD AVF/AVG patients, and a history of cancer was higher in the emergent-start HD-CVC patients, compared with their planned-start counterparts. Further, the emergent-start PD patients displayed a lower serum albumin and a higher phosphorus level than their planned-start counterparts. The emergent-start HD patients exhibited lower serum albumin and hemoglobin levels, and a higher phosphorus level than their planned-start counterparts, except they tended to have a higher estimated GFR (eGFR) at baseline.

**Table 1:** Baseline patient characteristics according to dialysis modality and initiation pattern for first time dialysis

	All	PD			HD			
		Planned-start	Emergent-start	*p* value	Planned-start	Emergent-start		*p* value
						AVF/AVG	CVC	
N	507	43	68	–	116	243	37	–
Men, n (%)	288 (56.8)	21 (48.8)	40 (58.8)	0.303^a^	65 (56.0)	140 (57.6)	22 (59.5)	0.925^a^
Age, years, n (%)	61.8 ± 15.5	52.1 ± 10.2	49.3 ± 13.3	0.327^b^	67.3 ± 13.2	63.8 ± 14.6	61.8 ± 15.5	0.075^d^
≧65	233 (44.0)	4 (9.3)	8 (11.8)	0.763^c^	69 (59.5)	119 (49.0)	23 (62.2)	0.092^a^
Predialysis education program	281 (55.4)	32 (74.4)	30 (44.1)	0.002^a^	106 (91.4)*^#^	100 (41.2)*	13 (35.1)^#^	< 0.001^a^
Primary renal diseases, n (%)								
Diabetes mellitus	217 (42.8)	10 (23.3)	17 (25.0)	0.835^a^	56 (48.3)	119 (49.0)	15 (40.5)	0.631^a^
Glomerulonephritis	115 (22.7)	16 (37.2)	30 (44.1)	0.472^a^	18 (15.5)	42 (17.3)	9 (24.3)	0.467^a^
Others	175 (34.5)	17 (39.5)	21 (30.9)	0.349^a^	42 (36.2)	82 (33.7)	13 (35.1)	0.898^a^
Comorbidities, n (%)								
Diabetes mellitus	247 (48.7)	12 (27.9)	19 (27.9)	0.997^a^	60 (51.7)	139 (57.2)	17 (45.9)	0.338^a^
Hypertension	426 (84.0)	38 (88.4)	56 (82.4)	0.391^a^	99 (85.3)	207 (85.2)	26 (70.3)	0.062^a^
Hyperlipidemia	117 (23.1)	9 (20.9)	23 (33.8)	0.144^a^	27 (23.3)	48 (19.8)	10 (27.0)	0.515^a^
CAD	65 (12.8)	4 (9.3)	9 (13.2)	0.763^c^	10 (8.6)	34 (14.0)	8 (21.6)	0.102^a^
CHF	80 (15.8)	3 (7.0)	11 (16.2)	0.241^c^	10 (8.6)*	50 (20.6)*	6 (16.2)	0.018^a^
VHD	25 (4.9)	1 (2.3)	2 (2.9)	1.000^c^	2 (1.7)	16 (6.6)	4 (10.8)	0.058^a^
CVA	44 (8.7)	2 (4.7)	1 (1.5)	0.558^c^	12 (10.3)	21 (8.6)	8 (21.6)	0.054^a^
PAOD	27 (5.3)	1 (2.3)	5 (7.4)	0.402^c^	5 (4.3)	14 (5.8)	2 (5.4)	0.848^a^
Cancer	44 (8.7)	2 (4.7)	1 (1.5)	0.558^c^	13 (11.2)^#^	19 (7.8)^$^	9 (24.3)^#$^	0.008^a^
Laboratory parameters								
Creatinine, mg/dl	12.3 ± 5.1	12.6 ± 4.0	15.4 ± 6.1	0.008^b^	11.2 ± 4.0	12.1 ± 5.1	12.3 ± 5.1	0.057^d^
eGFR, ml/min/1.73 m^2^	4.1 ± 1.8	3.9 ± 1.8	3.6 ± 2.0	0.111^b^	4.1 ± 1.6^#^	4.1 ± 1.8^$^	5.1 ± 2.2^#$^	0.020^d^
Albumin, g/dl	3.6 ± 0.6	4.1 ± 0.5	3.7 ± 0.5	0.002^b^	3.8 ± 0.5*^#^	3.4 ± 0.6*	3.6 ± 0.6^#^	< 0.001^d^
Hemoglobin, g/dl	8.7 ± 1.6	9.1 ± 1.4	8.4 ± 1.9	0.130^e^	9.3 ± 1.4*	8.3 ± 1.5*	8.7 ± 1.6	< 0.001^f^
Potassium, mmol/l	4.6 ± 0.9	4.5 ± 0.7	4.7 ± 0.9	0.365^b^	4.5 ± 0.8	4.7 ± 1.0	4.6 ± 0.9	0.395^d^
Phosphorus, mg/dl	6.7 ± 2.2	5.8 ± 1.5	7.7 ± 2.5	< 0.001^b^	5.9 ± 1.9*	6.8 ± 2.2*	6.7 ± 2.2	0.010^d^

*Abbreviations: CAD* coronary artery disease, *CHF* congestive heart failure, *VHD* valvular heart disease, *CVA* cerebrovascular accident, *PAOD* peripheral arterial occlusive disease. ^a^ χ^2^; ^b^ Mann-Whitney U test; ^c^ Fisher’s exact test; ^d^ Kruskal–Wallis H test; ^e^ t-test; ^f^ ANOVA. *^, #, $^ *p* < 0.05

### Factors associated with emergent initiation of dialysis

More than two-thirds of the participants started long-term dialysis following acute dialysis via a temporary HD catheter. The reasons (percentage) for emergent dialysis were as follows: fluid overload (38.2%), severe metabolic acidosis (19.1%), severe hyperkalemia (17.6%), serositis (8.8%), gastrointestinal bleeding (8.8%), and nausea/vomiting (7.4%). Because decisions to initiate acute dialysis were not made through a protocol-based process, we performed a multivariate logistic regression analysis to determine the predictors for being assigned to emergent-start dialysis. As shown in Table 2, the lack of predialysis education, along with a lower eGFR value and a lower serum albumin level, was more likely to be associated with initiation of acute dialysis via a temporary HD catheter in either PD or HD population.

**Table 2:** Multivariate logistic regression analysis showing predictors for emergent initiation of dialysis in incident ESRD patients

Emergent-start dialysis	PD		HD	
	Odds ratio (95% CI)	*p* value	Odds ratio (95% CI)	*p* value
Predialysis education program (no vs. yes)	3.17 (1.19–8.42)	0.021	24.77 (10.18–60.25)	< 0.001
eGFR (per ml/min/1.73 m^2^)	0.68 (0.51–0.92)	0.012	0.63 (0.53–0.75)	< 0.001
Albumin (per g/dl)	0.14 (0.04–0.43)	0.001	0.26 (0.15–0.46)	< 0.001
Comorbidities (yes vs. no)				
Coronary artery disease	–	–	2.87 (1.05–7.86)	0.040
Valvular heart disease	–	–	5.89 (1.17–29.78)	0.032

Variable selection for multivariate logistic regression model was performed using stepwise multiple regression (stepwise forward and backward selection method as *p* < 0.05 criterion). For PD patients, it showed a percentage of concordant pairs = 94.6%, adjusted generalized R^2^ = 0.261, estimated area under the receiver operating characteristic curve = 0.793, and Hosmer and Lemeshow goodness of fit test *p* = 0.853 > 0.05 (d.f = 8). The predicted probability of emergent-start PD = 1/(1 + exp.[− (8.93 + 1.153 x (predialysis education program) - 0.38 x (eGFR) - 1.99 x (albumin)]). For HD patients, it showed a percentage of concordant pairs = 91.2%, adjusted generalized R^2^ = 0.414, estimated area under the receiver operating characteristic curve = 0.888, and Hosmer and Lemeshow goodness of fit test *p* = 0.115 > 0.05 (d.f = 8). The predicted probability of emergent-start HD = 1/(1 + exp.[− (10.06 + 3.21 x (predialysis education program) - 0.46 x (eGFR) - 1.35 x (albumin) + 1.06 x (coronary artery disease) + 1.77 x (valvular heart disease)])

### Outcome distribution

The 2-year cumulative outcomes are shown in Fig. 1. Overall, 63 patients (12.4%) died from any cause, 9 (1.8%) received renal transplantation, 343 (67.7%) remained under HD (including 15 switched from initial PD), and 92 (18.1%) remained under PD (including 4 switched from initial HD). As shown in Table 3, there were no differences in the crude incidence for mortality rates (all-cause and cause-specific mortality) or peritonitis between the planned-start and the emergent-start PD patients, although the rate of overall hospitalization was lower in the planned-start group. In contrast, the emergent-start HD-CVC patients, compared with their planned-start and emergent-start HD-AVF/AVG counterparts, displayed a higher rate of overall death, access infection-related mortality and overall hospitalization.

**Table 3:** Unadjusted cumulative incidence for all-cause and cause-specific mortality, overall hospitalization and peritonitis

	PD			HD			
	Planned-start	Emergent-start	*p* value	Planned-start	Emergent-start		*p* value
					AVF/AVG	CVC	
*All-cause mortality*, n (%)	2 (4.6)	3 (4.5)	1.000^a^	9 (7.7)^#^	34 (14.0)^$^	16 (43.2)^#$^	< 0.001^b^
*Cause-specific mortality, n (%)*							
Infection	1 (2.3)	0 (0)	0.387^a^	5 (4.3)^#^	18 (7.9)^$^	9 (24.3)^#$^	< 0.001^b^
Peritonitis related	1 (2.3)	0 (0)	0.387^a^	–	–	–	
HD access related	–	–	–	2 (1.7)^#^	3 (1.2)^$^	6 (16.2)^#$^	< 0.001^b^
Other infection	0 (0)	0 (0)	–	3 (2.6)	16 (6.7)	3 (8.1)	0.235^b^
Cardiovascular	0 (0)	1 (1.5)	1.000^a^	2 (1.7)	9 (3.7)	1 (2.7)	0.588^b^
Cancer	1 (2.3)	1 (1.5)	1.000^a^	0 (0)^#^	3 (1.2)^$^	4 (10.8)^#$^	< 0.001^b^
Others	0 (0)	1 (1.5)	1.000^a^	2 (1.7)	3 (1.2)	2 (5.4)	0.200^b^
*Overall hospitalization, n (%)*	18 (41.9)	42 (61.8)	0.040^b^	50 (43.1)*^#^	136 (56.0)*	23 (62.2)^#^	0.036^b^
Hospital days, per admission^c^	5.5 (3.3–10.5)	6.9 (4.0–11.6)	0.545^d^	7.1 (4.0–13.7)	7.8 (4.0–15.7)	12.0 (5.8–25.9)	0.268^e^
Hospital admission rate (per 100 patient-months)	5.52	7.35	< 0.001^f^	5.01^#^	5.38^$^	13.87^#$^	< 0.001^f^
*Incidence of peritonitis* (per 100 patient-months)	0.97	1.29	0.937^f^	–	–	–	–

^a^Fisher’s exact test; ^b^ χ^2^; ^c^ Values reported as median (interquartile range); ^d^ Mann-Whitney U test; ^e^ Kruskal-Wallis H test; ^f^ Poisson test. *^, #, $^ *p* < 0.05

### Factors associated with patient outcomes

**Fig. 2:**
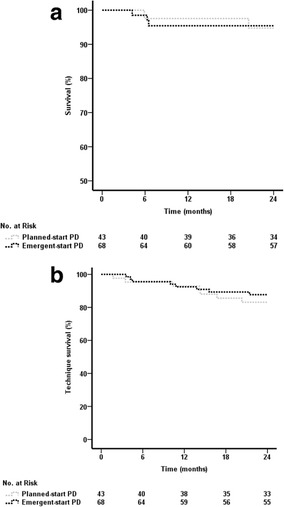
**a** Survival between patients undergoing emergent-start PD and planned-start PD (Log-Rank test, χ^2^ = 0.005, *p* = 0.941); **b** Technique survival between patients undergoing emergent-start PD and planned-start PD (Log-Rank test, χ^2^ = 0.406, *p* = 0.524). PD, peritoneal dialysis

Unadjusted cumulative incidence curves using the Kaplan–Meier method revealed no difference in all-cause mortality (Fig. 2a) or technique failure (Fig. 2b) between the planned-start and the emergent-start PD patients. The propensity score-adjusted, multivariate Cox regression model, which treated dialysis modality or access type as a time-dependent variable, confirmed the lack of differences in the risk of overall death between the 2 subgroups (Table 4). Likewise, the Cox regression model taking into account the competing risks of death or transplantation confirmed the absence of differences in the risk of technique failure (Table 5). In contrast, the concurrent emergent-start HD-CVC patients displayed a greater risk of overall death compared with the planned-start and the emergent-start HD-AVF/AVG counterparts (Table 4, models 1 and 2, respectively). Further propensity score-adjusted Cox regression models taken into account the competing events of death from other cause showed a significantly lower risk of access infection-related mortality in the planned-start HD-AVF/AVG and emergent-start HD-AVF/AVG patients than the emergent-start HD-CVC counterparts [sub-HR 0.04 (95% CI 0.002–0.62), *P* = 0.022; sub-HR 0.06 (95% CI 0.01–0.29), *P* < 0.001, respectively].

**Table 4:** Time-dependent Cox proportional hazards models showing predictors for all-cause mortality by dialysis modality, adjusted for the propensity score

Parameters	PD		Parameters	HD			
	Hazard ratio (95% CI)	*p* value		Model 1		Model 2	
				Hazard ratio (95% CI)	*p* value	Hazard ratio (95% CI)	*p* value
Propensity score	0.13 (0.003–5.22)	0.281	Propensity scores	0.34 (0.07–1.59)	0.168	3.71 (0.54–25.76)	0.185
Planned-start PD vs. Emergent-start PD	0.70 (0.10–4.82)	0.713	Planned-start HD vs. Emergent-start HD-CVC	0.045 (0.01–0.17)	< 0.001	–	–
–	–	–	Emergent-start HD-AVF/AVG vs. Emergent-start HD-CVC	–	–	0.31 (0.14–0.68)	0.003
Age (≧65 vs. < 65 years)	5.209 (0.91–29.90)	0.064	Age (≧65 vs. < 65 years)	4.275 (1.18–15.47)	0.027	4.58 (1.99–10.53)	< 0.001
			Comorbidity (yes vs. no)				
–	–	–	Hyperlipidemia	0.09 (0.005–0.61)	0.018	–	–

Variable selection for Cox regression hazards modeling was performed using stepwise forward and backward selection method as *p* < 0.15 criterion. Model 1 included the Planned-start HD and Emergent-start HD-CVC subgroups, while Model 2 included the Emergent-start HD-AVF/AVG and Emergent-start HD-CVC subgroups. Both models were adjusted for sex, predialysis education program, eGFR, albumin, hemoglobin, and all comorbidities

**Table 5:** Competing-risks Cox regression models showing predictors for technique failure in PD patients, adjusted for the propensity score

Parameters	PD	
	Sub-hazard ratio (95% CI)	*p* value
Propensity score	6.09 (0.41–90.77)	0.19
Planned-start PD vs. Emergent-start PD	1.72 (0.70–4.25)	0.24
Sex (female vs. male)	3.75 (1.15–12.25)	0.03
Comorbidity (yes vs. no)		
Diabetes mellitus	6.72 (1.91–23.69)	0.003

Variable selection for Cox regression hazards modeling was performed using stepwise forward and backward selection method as *p* < 0.15 criterion. The model was adjusted for age, predialysis education program, eGFR, albumin, hemoglobin, and all comorbidities

Table 6 shows the results of the propensity score-adjusted Cox regression models for all-cause hospitalization taken into account the competing events of death and transplantation. No differences were detected in the risk of overall hospital admission between the 2 subgroups of PD. The presence of vascular disorders [coronary artery disease and peripheral arterial occlusive disease (PAOD)] were associated with a greater likelihood of overall hospitalization.

**Table 6:** Competing-risks Cox regression models showing predictors for overall hospitalization in PD patients, adjusted for the propensity score

Parameters	PD	
	Sub-hazard ratio (95% CI)	*p* value
Propensity score	1.79 (0.42–7.66)	0.431
Planned-start PD vs. Emergent-start PD	0.80 (0.45–1.43)	0.459
Comorbidity (yes vs. no)		
Coronary artery disease	2.21 (1.16–4.20)	0.016
PAOD	5.00 (2.82–8.88)	<0.001

*Abbreviations: PAOD* peripheral arterial occlusive disease

Variable selection for Cox regression hazards modeling was performed using stepwise forward and backward selection method as *p* < 0.15 criterion. The model was adjusted for age, sex, predialysis education program, eGFR, albumin, hemoglobin, and all comorbidities

## Discussion

This study provides evidence showing emergent starters of PD had similar risks of patient survival, technique failure and overall hospitalization, compared with the planned-start counterparts. The ‘intent-to-defer’ strategy as recommended by current clinical practice guidelines [20] may lead to uncertainties in the timing of dialysis access creation with respect to dialysis initiation. Our previous report has shown that late-stage CKD patients who underwent planned but urgent start of long-term HD fare the worst among a prospective cohort of incident HD patients [14]. By contrast, the present study shows that emergent starters of PD exhibited similar patient outcomes compared with the planned starters. This clinical scenario is somewhat reminescent of the resurgent momentum of urgent-start PD program, in which patients starting PD within 2 weeks after placement of the PD catheter exhibited outcomes comparable to the traditionally planned counterpart [5, 7, 8]. It is advocated that urgent-start PD be a sound treatment option for late-referred CKD patients who do not have a mature vascular access at the time of dialysis initiation [6, 10, 21]. Similarly, data accrued from this study suggest that post-initiation PD can be a relatively safe long-term dialysis treatment for late-referred CKD patients, given the poor outcome of the concurrent emergent-start HD-CVC patients who commenced long-term dialysis with a tunneled cuffed CVC.

For emergent-start HD-AVF/AVG patients, the longer waiting time for the maturation of post-initiation AVFs, compared with placement of a PD catheter, might be associated with prolonged use of a temporary HD catheter. This could lead to an increased risk of catheter-related bloodstream infections [22, 23]. In that context, post-initiation PD may be a more convenient alternative to continue long-term dialysis within a shorter period of waiting time [13, 24]. Our observations that the emergent-start PD patients showed a technique survival comparable to that of the traditionally planned-start PD counterparts lend further support to the choice of PD, as opposed to HD with a tunneled cuffed CVC, following emergent dialysis via a temporary HD catheter.

In the present study, we found patients without predialysis education/care were more likely to be assigned to emergent dialysis via a temporary HD catheter. The exact reason for this phenomenon was not clear but could be related in part to lack of beneficial effects of multidisciplinary predialysis care which included protection from emergent initiation of dialysis [25–27]. Besides lack of predialysis care, we observed that patients with a lower baseline eGFR and serum albumin level were more prone to emergent-start dialysis. Theoretically, the lower eGFR at dialysis initiation reflected more advanced CKD. Therefore, these patients might suffer more severe CKD complications secondary to hypoalbuminemia [28, 29]. Although our data showed no survival disadvantage in emergent-start PD patients compared to their planned counterparts, we surmise that more effective strategies to identify barriers of planned-start PD should be employed so that emergent-start PD and the associated poor nutritional status can be prevented.

As have been reported elsewhere, certain comorbid illnesses are well known to exert a negative impact on patient outcomes after diagnosis of ESRD [30, 31]. In our PD population, the presence of vascular disorders, e.g., coronary artery disease and PAOD, was associated with a greater likelihood of overall hospitalization. This finding coincided with prior reports that showed previous cardiovascular event was a poor prognostic factor for all-cause hospitalization in PD patients [32, 33]. Likewise, concomitant PAOD is associated with increased risk of amputations and foot-related hospital admission and mortality in both PD and HD patients [34, 35]. Taken together, it would be appropriate that new-onset ESRD patients harboring the above-mentioned clinical conditions be guarded by more meticulous care so that early hospitalization can be prevented after commencing long-term PD treatment.

This observational study is unique in that it included all new-onset ESRD patients during the same time frame, thus avoiding a selection bias that might have occurred if we chose to analyze only the PD population while ignoring the concurrent HD patients. There existed other potential biases in the analysis of this study. The non-randomized allocation of patients could have led to a misclassification bias. Nevertheless, we tried to minimize such inclination by constructing the propensity score for being assigned to emergent-start dialysis and included it for all subsequent survival analyses. Further, the dialysis modality or access type for any individual patient might not be constant through the observation period. Therefore, we performed time-dependent Cox regression analysis to adjust for the time-varying nature of this particular variable. We also adjusted for the competing-risk confounding of death or transplantation when modeling such outcomes as technique failure and overall hospitalization. However, despite these efforts, we could not exclude the possibility of residual confounding, e.g., the impact of baseline urine output. Future research with a randomized design and a larger sample size may be required to validate the observations accrued from this study.

## Conclusion

In conclusion, this observational study shows that in late-referred CKD patients who have undergone emergent dialysis via a temporary HD catheter, PD can be a safe and effective long-term treatment option. Nevertheless, due to the potential complications and cost concerns, such practice of PD initiation would better be replaced with a planned-start mode by employing more effective predialysis therapeutic education and timely catheter placement.
